# Targeting UBR5 in hepatocellular carcinoma cells and precise treatment via echinacoside nanodelivery

**DOI:** 10.1186/s11658-022-00394-w

**Published:** 2022-10-12

**Authors:** Menghan Wang, Xing Ma, Guoyu Wang, Yanan Song, Miao Zhang, Zhongchao Mai, Borong Zhou, Ying Ye, Wei Xia

**Affiliations:** 1 Department of Nuclear Medicine, The Seventh People’s Hospital, Shanghai University of Traditional Chinese Medicine, 358 Datong Rd, Pudong New Area, Shanghai, 200137 China; 2 Central Laboratory, The Seventh People’s Hospital, Shanghai University of Traditional Chinese Medicine, 358 Datong Rd, Pudong New Area, Shanghai, 200137 China

**Keywords:** Nanoparticle, Echinacoside, Drug delivery, Hepatocellular carcinoma, UBR5

## Abstract

**Background:**

Hepatocellular carcinoma (HCC) is among the most common and malignant cancers with no effective therapeutic approaches. Echinacoside (ECH), a phenylethanoid glycoside isolated from Chinese herbal medicine, *Cistanche salsa*, can inhibit HCC progression; however, poor absorption and low bioavailability limit its biological applications.

**Methods:**

To improve ECH sensitivity to HepG2 cells, we developed a mesoporous silica nanoparticle (MSN)-based drug delivery system to deliver ECH to HepG2 cells via galactose (GAL) and poly(ethylene glycol) diglycidyl ether (PEGDE) conjugation (ECH@Au@MSN-PEGDE-GAL, or ECH@AMPG). Gain- and loss-of-function assays were conducted to assess the effects of UBR5 on HCC cell apoptosis and glycolysis. Moreover, the interactions among intermediate products were also investigated to elucidate the mechanisms by which UBR5 functions.

**Results:**

The present study showed that ubiquitin protein ligase E3 component N-recognin 5 (UBR5) acted as an oncogene in HCC tissues and that its expression was inhibited by ECH. AMPG showed a high drug loading property and a slow and sustained release pattern over time. Moreover, owing to the valid drug accumulation, ECH@AMPG promoted apoptosis and inhibited glycolysis of HepG2 cells in vitro. In vivo experiments demonstrated that AMPG also enhanced the antitumor effects of ECH in HepG2 cell-bearing mice.

**Conclusions:**

Our results indicated the clinical significance of UBR5 as a therapeutic target. On the basis of the nontoxic and high drug-loading capabilities of AMPG, ECH@AMPG presented better effects on HCC cells compared with free ECH, indicating its potential for the chemotherapy of HCC.

**Supplementary Information:**

The online version contains supplementary material available at 10.1186/s11658-022-00394-w.

## Background

Hepatocellular carcinoma (HCC), a liver malignancy found in patients with persistent liver disease and cirrhosis, is one of the three most common causes of cancer-related death worldwide and the fifth most prominent malignant cancer [1, 2]. Patients with HCC have limited treatment options, including tumor resection, ablation, chemotherapy, liver transplant, and radiotherapy [3]. Moreover, patients with advanced HCC have a low survival rate (> 5 years) [4]. Therefore, there is a need for a deeper understanding of the molecular mechanisms underlying HCC progression to develop better treatment interventions against HCC. Ubiquitin protein ligase E3 regulates various important biological processes, particularly cell differentiation, colony formation, proliferation, apoptosis, and immunity [5, 6]. However, dysregulation of ubiquitin protein ligase E3 can lead to tumor development and metastasis [7]. As an E3 ubiquitin ligase, ubiquitin protein ligase E3 component N-recognin 5 (UBR5) regulates cell proliferation, cell maturation, glycolysis, immune response, and chemoresistance, and its atypical cellular levels are linked to various human diseases, including cancer [8–12]. UBR5 dysregulation can promote oncogenesis of multiple types of cancer, and UBR5 may have the potential to act as a prognostic biomarker for patients with cancer. C2orf40 can inhibit HCC through its interaction with UBR5 [13], suggesting that UBR5 may be a potential therapeutic target in HCC. Luteolin, a natural flavonoid, can inhibit the PI3K/AKT pathway, thereby decreasing UBR5 expression in esophageal cancer cells [14]. However, no potent UBR5 inhibitor has been used for the treatment of HCC.

Several natural compounds extracted from Chinese herbs have shown anticancer activity via inhibition of cancer angiogenesis, metastasis, and proliferation [15, 16]. Echinacoside (ECH), an active constituent of *Cistanche tubulosa*, shows strong antiproliferative and pro-apoptotic activities in various types of cancer, including HCC [15, 17]. However, despite ECH possessing many pharmacological properties, orally administered ECH has poor absorption and low bioavailability [18, 19]. In addition, side effects also limit the clinical use of ECH. To address this problem, nanoparticle-based delivery systems with targeting capability have been developed to ferry chemotherapeutic drugs from the injection site to their intracellular targets in cancer cells via the enhanced permeability and retention effect [20]. Hydrophilic polymers, such as poly(ethylene glycol) (PEG), have been attached to the surface of nanosystems to prevent the recognition of serum proteins and bypass phagocytic clearance from the circulation [21]. Several strategies for targeted delivery to cancer cells or animal xenografts have recently been developed, such as the use of mesoporous silica nanoparticles (MSNs) [22]. MSNs are classic carrier materials with ordered pores, high pore volume, high surface area, and dense distribution of silanol groups at the surface. The surface silanol groups facilitate the achievement of subsequent functionalization processes. These features make MSMs ideal candidates for drug delivery system applications [23]. In this work, MSNs cross-linked with poly(ethylene glycol) (diglycidyl) ether (PEGDE) and coated with galactose could activate targeted internalization by cancer cells, providing precise delivery of ECH to tumors for improved therapeutic outcomes with reduced side effects and increased chemosensitivity.

## Materials and methods

### Chemicals

Cetyltrimethyl ammonium bromide (CTAB), sodium borohydride (NaBH_4_), tetraethyl orthosilicate (TEOS), ascorbic acid, PEGDE (molecular weight ~ 526), GAL, dimethyl sulfoxide (DMSO), ethanol, methanol, and tetrachloroauric acid (HAuCl_4_·3H_2_O) were purchased from Sigma-Aldrich (St. Louis, MO, USA). ECH (purity: ≥ 99.85%) was purchased from MedChemExpress (Monmouth Junction, NJ, USA; HY-N0020).

### Bioinformatics analysis

We extracted gene expression and DNA copy-number data from The Cancer Genome Atlas (TCGA) database for HCC, comprising 371 tumor tissue cases and 50 healthy liver tissue cases. We assessed the effect of UBR5 copy-number variation (CNV) on its expression using the one-sided Jonckheere–Terpstra test. In addition, we applied gene set enrichment analysis (GSEA) algorithm to identify pathways with significant upregulation (high versus low UBR5 expression). We analyzed seven UBR family genes in patients with HCC from the TCGA database to evaluate the function of ubiquitin protein ligase E3, especially the UBR family genes, in HCC.

### Clinical samples

This study included two cohorts of patients with HCC admitted to hospitals from March 2019 to October 2021. Cohort 1 comprised 25 fresh tumor tissues and paired normal tissues, whereas cohort 2 comprised 250 formalin-fixed, paraffin-embedded tumor and 30 adjacent normal tissues. All patients provided written informed consent prior to study inclusion. The protocols for cancer specimen retrieval were approved by the medical ethics commission of The Seventh People’s Hospital (approval number 2018-HIRB-045, date: 15 October 2018).

### DNA CNV detection

The Tguide S32 Magnetic Tissue Genomic DNA Kit (TIANGEN Biotech, Beijing, China) was used to extract DNA from samples. The QX200 Droplet Digital PCR Assay was used to detect *UBR5* gene copy numbers, as previously described [24].

### Immunohistochemistry

Tumor and adjacent normal tissues in cohort 2 were used for immunohistochemistry (IHC) analysis. A standard protocol using anti-UBR5 antibody (ab70311; Abcam, Cambridge, MA, USA) was used to detect UBR5 expression. Sections were counterstained with hematoxylin, and immunoreactivity was scored using the H-score system by two investigators on the basis of the proportion of positively stained cells. Patients with ≥ 50% positive cells were graded as highly expressive, whereas those with < 50% positive cells were classified as lowly expressive.

### Cell culture and transfection

Human HepG2 and Huh7 cells (ATCC, Gaithersburg, MD, USA) and human liver cell lines LO2 (Cell Bank, Chinese Academy of Sciences, Shanghai, China) were cultured in Dulbecco’s modified Eagle medium augmented with 10% fetal bovine serum and 1% penicillin/streptomycin (Life Technologies, Carlsbad, CA, USA) at 37 °C under 5% CO_2_ and 95% relative humidity.

UBR5 and AXIN1 amplicons were cloned into the pLVX-Puro plasmid (Clontech Laboratories, Mountain View, CA, USA), and the synthetic UBR5 short hairpin RNA (shRNA) sequence was inserted into a linearized pLKO.1 plasmid (Addgene, Watertown, MA, USA). The recombined plasmids, including psPAX2 and pMD2G (packaging plasmids), were co-transfected into 293T cells with Lipofectamine 2000 reagent (Invitrogen, Carlsbad, CA, USA). The recombined proteins were then harvested for cell transduction 48 h after transfection. Cells with blank pLVX-Puro or pLKO.1-scramble shRNA were used as negative controls.

### ECH@AMPG synthesis

#### Synthesis of Au@MSN

Au@MSN was synthesized using the sol–gel method as previously described with slight modifications [25]. First, we prepared CTAB-capped Au seeds via HAuCl_4_ reduction using NaBH_4_. We stirred 7.5 mL of 0.1 M CTAB solution with 250 μL of 10 mM HAuCl_4_ and added water to reach a volume of 9.4 mL. Next, we added 0.6 mL of ice-cold aqueous 0.01 M NaBH_4_ solution to the mixture. The seeds that immediately formed were used within 2–5 h. The growth suspension/solution for Au nanoparticles (NPs) contained 50 mL of 0.1 M CTAB, 2.5 mL of 0.01 M HAuCl_4_, 1 mL of 0.5 M H_2_SO_4_, and 400 μL of 0.1 M ascorbic acid. Growth was initiated by adding 120 μL of seeds, with a constant growth medium temperature of 30 °C throughout the procedure. The as-synthesized Au NPs were washed thrice by centrifugation, the residue was diluted with 20 mL of water, and 200 μL of 0.1 M NaOH was added with stirring. Subsequently, we added three 60 μL doses of 20% TEOS in methanol with mild stirring at 30 min intervals, incubated the mixture at 26–28 °C to react for 3 days, and then separated the mixture by centrifugation thrice with ethanol washing. The obtained product was oven dried for 24 h at 60 °C to evaporate the ethanol. To extract CTAB from the obtained product, we added a 100 μL aliquot of HCl to 100 mL of ethanol solution with 0.5 g of the obtained product, stirred the mixture for 8 h at 60 °C, and centrifuged it to harvest the final product, which was later oven dried at 60 °C. At this stage, the product was called Au@MSN (AM).

#### Grafting of PEGDE to AM

The AM was filtered, washed thrice with a large amount of methyl isobutyl ketone, and centrifuged. After centrifugation, the total volume was adjusted with methyl isobutyl ketone to 2 mL. Next, we added 5 mL of PEGDE with a small amount of stannous chloride (0.1 wt%) as a catalyst, stirring magnetically. Under the protection of a nitrogen atmosphere, the mixed solution was heated for 4 h at 100 °C; the obtained product was cooled down, filtered, and rinsed with a sufficient amount of methyl isobutyl ketone; and the final product of this stage was vacuum oven dried overnight at 60 °C to recover AM-PEGDE (AMP).

#### Conjugation of galactose to the AMP surface

We added 1.0 g of AMP and 0.1 g of GAL to 50 mL of DMSO and quickly stirred the mixture to facilitate dispersion. Next, the mixture was again stirred for 4 h at room temperature using a magnetic stirrer and subsequently filtered, after which it was washed with sufficient DMSO and then methanol. The obtained AMP-GAL (AMPG) was vacuum oven dried overnight at 60 °C.

#### ECH loading to AMPG

We incubated 100 mg of AMPG in 100.0 mL (2.5 mg mL^−1^) of ECH for 72 h at 50 °C. The unbound excess ECH was removed from the supernatant by centrifugation at 12,000 rpm for 15 min. The obtained ECH@AMPG was freeze-dried for later use.

### Characterization

Images were obtained using a JEM-2100F transmission electron microscope (TEM; JEOL Ltd., Tokyo, Japan). The Brunauer–Emmett–Teller surface area, N_2_ adsorption–desorption isotherms, and pore volume were obtained using the Micrometritics ASAP 2010 instrument (Micrometritics, Norcross, GA, USA). Fourier transform infrared (FT-IR) spectra were acquired using the Nicolet iS10 instrument (Thermo Fisher Scientific, Waltham, MA, USA). In addition, thermogravimetric analysis (TGA) was performed using the STA449C thermal analysis instrument (Netzsch, Germany). High-performance liquid chromatography (HPLC) was performed using the Agilent HP1100 instrument (Agilent Technologies, Santa Clara, CA, USA). Dynamic light scattering (DLS) was performed using the TC type Dynapro Titan instrument (Wyatt Technology, Santa Barbara, CA, USA).

### ECH release in vitro

We suspended three 10-mg consignments of ECH@AMPG in 10 mL of phosphate-buffered saline (PBS) buffer (pH 4, 5.6, and 7.4). The suspensions were dialyzed in dialysis bags permeable to particles of up to 3000 Da molecular weight and then immersed in a beaker containing 100 mL of PBS buffer at corresponding pH values. The dissolution medium was sustained at 100 mL with perpetual mixing at 37 °C. At a scheduled time, we collected 1 mL of PBS buffer for HPLC measurement to calculate the amount of ECH release.

### CCK-8 assay

We characterized cellular proliferation using the Cell Counting Kit 8 (CCK-8; SAB Biotech., College Park, MD, USA). HepG2 and LO2 cells were cultured in 96-well plates at a density of 3 × 10^3^ cells per well for 12 h. Next, the cells were treated with different concentrations of ECH (0, 1, 2, 5, 10, or 20 μg/mL). After periodic incubation for 0, 12, 24, and 48 h, CCK-8 reagent was added to each well and the cells were again incubated for 1 h. Finally, absorbance was measured for each well at a wavelength of 450 nm.

### Cellular uptake behavior

Confocal laser scanning microscopy (CLSM) was used to evaluate the cellular uptake ability of AMPG by HepG2 cells. HepG2 cells were seeded in a 24-well plate at a density of 3 × 10^4^ cells per well and incubated with 5 μg/mL of Cy7-labeled AMPG or AM as a control. After incubation for 6 h, the nuclei, cytoskeleton, and AM or AMPG were stained with 4′,6-diamidino-2-phenylindole (DAPI; Beyotime Biotechnology, Shanghai, China), fluorescein isothiocyanate (FITC)-phalloidin (Abcam), and Cy7 (MedChemExpress) for 40 min, respectively, and observed via CLSM at 405, 562, and 488 nm laser excitation. TEM detection for samples fixed with 4% glutaraldehyde was performed as previously described [26].

### Cell apoptosis assay

Adherent HepG2 cells were seeded in a six-well plate at a density of 5 × 10^5^ cells per well and allowed to grow until 50% confluence. Flow cytometry was used to evaluate cellular apoptosis. For staining, the cells were incubated for 15 min in the dark at 4 °C with 5 μL of FITC-labeled recombinant annexin V (annexin V–FITC), followed by incubation for a further 15 min with 5 μL of propidium iodide. Cellular apoptosis was profiled using the FACScan flow cytometer (Becton Dickinson, Franklin Lakes, NJ) with Cell Quest software (Becton Dickinson).

To further examine the role of UBR5 and the effect of ECH@AMPG on UBR5-mediated tumor progression, HepG2 cells were transduced with UBR5 expression vector and treated with free ECH, AMPG, and ECH@AMPG. The HepG2 cell apoptosis rate was measured before and after changing UBR5 expression.

### Extracellular flux analysis

The cellular oxygen consumption rate (OCR) and extracellular acidification rate (ECAR) were phenotyped using the Seahorse XF24 Extracellular Flux Analyzer as previously described [27].

### Co-immunoprecipitation and ubiquitination assay

The target protein was incubated for 1 h with Protein A/G PLUS-Agarose, followed by incubation with anti-UBR5 antibody, anti-AXIN1 antibody, or normal IgG overnight at 4 °C. Protein A Sepharose was used to pull down the immunocomplexes, which were then analyzed by western blotting using anti-UBR5, anti-AXIN1, or anti-ubiquitin antibody.

### Quantitative reverse-transcription polymerase chain reaction (qRT-PCR)

We extracted total RNA from HCC tissues and HepG2 cells using TRIzol reagent (Invitrogen), followed by complementary DNA reverse transcription using the PrimeScript RT Reagent Kit with genomic DNA Eraser (TaKaRa Bio, Japan). qRT-PCR was performed using SYBR Premix Ex Taq GC (TaKaRa Bio) with the following forward and reverse primers: UBR5: 5′-ATCTACTTTATCGCCTGCTCAC-3′ and 5′-CAATGCTCCACCGTCTGC-3′; AXIN1: 5′-TGCCGACTTGCTGGACTTCTGG-3′ and 5′-TCTTGGTGGCTGGCTTGGTCTG-3′; GAPDH: 5′-AATCCCATCACCATCTTC-3′, and 5′-AGGCTGTTGTCATACTTC-3′. To calculate the fold-change in the mRNA expression of UBR5 and AXIN1, we used the 2^−ΔΔCT^ method.

### Western blotting

Whole protein was extracted using radioimmunoprecipitation assay lysis buffer (Beyotime) and quantified using the bicinchoninic acid Protein Assay Kit (Beyotime). Briefly, a 20 μg protein aliquot was loaded into 10% sodium dodecyl sulfate polyacrylamide gel electrophoresis for separation, followed by electroblotting on a polyvinylidene fluoride membrane (Merck Millipore, Burlington, MA, USA), blocking using 5% fat-free milk, and overnight incubation with the following primary antibodies: anti-UBR5 (ab70311; Abcam), anti-AXIN1 (ab55906; Abcam), anti-β-catenin (ab32572; Abcam), anti-Survivin (ab76424; Abcam), anti-C-myc (ab39688; Abcam), anti-lactate dehydrogenase A (LDHA, ab101562; Abcam), anti-pyruvate kinase isozymes M2 (PKM2, ab137852; Abcam), anti-H3 (ab1791; Abcam), and anti-GAPDH antibody (60004-1-1G; ProteinTech, Rosemont, IL, USA). They were washed thrice with Tris-buffered saline with Tween 20 and incubated with horseradish peroxidase-conjugated secondary antibodies (A0208, A0216; Beyotime). Finally, the blot was imaged using the Enhanced Chemiluminescence Detection Kit (Pierce Biotechnology), and the band intensity was measured using Image-Pro Plus 6.0 software, with GAPDH as the loading control.

### Targeting effect of AMPG in vivo

To verify the targeting effect of AMPG, a xenograft model constructed via subcutaneous administration of HepG2 cells was established. We detected the targeting effect of intravenously injected ECH@AMPG on tumor cells in vivo by using a xenograft tumor model through subcutaneous injection of HepG2 cells. Briefly, 1 × 10^6^ HepG2 cells were intravenously injected into the left posterior flank of 4-week-old BALB/c nude mice (401; Beijing Vital River Laboratory Animal Technology Co., Ltd., Beijing, China). After tumor formation for 1 week, Cy7-labeled AMPG loaded with or without ECH was injected into the mice via the tail vein at a dose of 5 mg/kg/day for 4 weeks. AMPG fluorescent images were obtained using the IVIS 200 system (PerkinElmer, Waltham, MA, USA) at 3, 6, 12, and 24 h to verify the AMPG biodistribution. In vivo surviving HepG2 cells were detected using bioluminescence at 0, 1, 2, 3, and 4 weeks to verify the in vivo targeting efficacy of AMPG. After intravenous injection, the mice (*n* = 8 per each group) were euthanized, and their key organs (liver, heart, lungs, kidneys, and spleen) and xenograft tumors were harvested, washed, fixed with 4% paraformaldehyde, and processed to produce paraffin-embedded sections. To evaluate toxicity and apoptosis, organ and tumor sections were stained with hematoxylin and eosin (H&E) or terminal deoxynucleotidyl transferase dUTP nick end labeling (TUNEL). IHC staining was performed to detect the UBR5 and AXIN1 expression in tumor xenograft using anti-UBR5 antibody (ab70311; Abcam) and anti-AXIN1 antibody (ab115205; Abcam), respectively. All protocols involving animals were reviewed and approved by the Animal Ethics Board of The Seventh People’s Hospital (approval number 2018-31, date: 15 October 2018). The animals were anesthetized via inhalation of 3% isoflurane and sacrificed via cervical dislocation on day 28.

## Statistical analysis

Statistical analyses were performed using GraphPad Prism version 8.4.2. Data from independent triplicates are shown as mean ± standard deviation. One-way analysis of variance and two-sided Student’s *t*-test were performed to compare different experimental groups. The overall survival of the patients was calculated using Kaplan–Meier analysis and Cox’s proportional hazards regression model. Differences among groups were analyzed using the log-rank test. *P* < 0.05 indicated statistical significance.

## Results

### UBR5 genomic amplification is dominant in HCC and is correlated with low patient survival

**Fig. 1:**
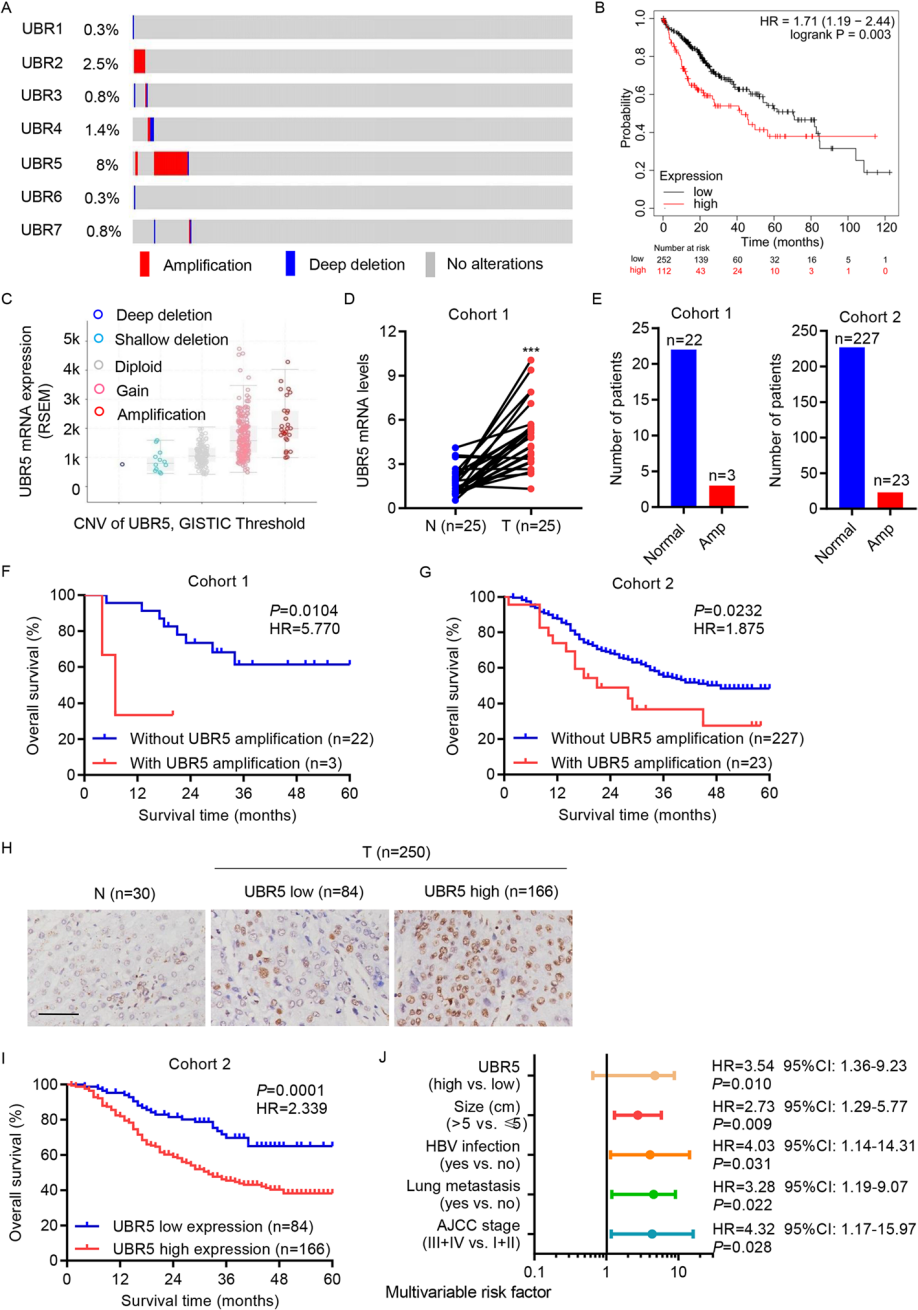
UBR5 genomic amplification is prevalent in HCC and is linked to poor prognosis. **A** CNV analysis of UBR family genes in patients with HCC from the TCGA database. **B** Overall survival of patients with HCC from the TCGA database. **C** UBR5 mRNA levels in patients with HCC with UBR5 CNVs from the TCGA database. **D** UBR5 mRNA levels in HCC tissues and paired normal liver tissue (*n* = 25) from cohort 1 was measured by qRT-PCR. **E** Statistical analysis of UBR5 copy number in cohorts 1 and 2. **F**, **G** Survival outcomes of patients with HCC with or without UBR5 amplification in cohorts 1 and 2. **H** IHC was used to characterize UBR5 expression in HCC (*n* = 250) and normal liver (*n* = 30) tissue in cohort 2. Scale bar, 100 μm. **I** Survival outcome of patients with HCC with high or low UBR5 expression in cohort 2. **J** Multivariate regression analysis was performed in cohort 2. ***P < 0.001 compared with N. *N* normal liver tissues; *T* HCC tissues

All UBR family genes were upregulated in HCC tissues (Additional file 1: Fig. S1A). DNA CNV is a metric of genomic variation, which is a common phenomenon among humans. Studies have attempted to identify CNVs linked to susceptibility or resistance to diseases, including cancer [28, 29]. CNV analysis showed that *UBR2*, *UBR4*, and *UBR5* were positive for copy-number amplification in patients with HCC from the TCGA database (amplification frequency > 1%), with *UBR5* significantly showing the maximum gain (Fig. 1A). High UBR5 expression led to poorer prognosis in patients with HCC from the TCGA database (Fig. 1B). Patients with UBR5-gained CNV had significantly higher UBR5 mRNA expression compared with those without CNV from the TCGA database (Fig. 1C). In cohort 1, tumor samples also had significantly higher UBR5 mRNA expression compared with normal samples (Fig. 1D).

In addition, UBR5 amplification was positive in 3 of the 25 (13.6%) patients in cohort 1 and 23 of the 250 (9.2%) patients in cohort 2 (Fig. 1E). Among patients with HCC in cohorts 1 and 2, those without UBR5 amplification showed significantly better survival outcomes than those with UBR5 amplification (Fig. 1F and G), indicating that copy numbers contribute to high UBR5 expression and poor survival outcomes in patients with HCC. UBR5 expression patterns in cohort 2 were measured using IHC analysis (Fig. 1H). Kaplan–Meier analysis showed that high UBR5 expression promoted poor prognosis in cohort 2 (Fig. 1I). A comparison between UBR5 expression and different clinicopathological features in cohort 2 showed that UBR5 expression was positively correlated with tumor size, American Joint Committee on Cancer (AJCC) stage, histological grade, and lung metastasis (Table 1) but not with sex, age, differentiation, and hepatitis B virus (HBV) infection. Multivariate regression analysis showed that UBR5 expression was a prognosticator of HCC aggressiveness, with a significant hazard ratio for envisaging clinical outcomes (Fig. 1J).

**Table 1:** Correlation of the expression of UBR5 with clinicopathologic features of patients with HCC

Clinicopathological parameter	UBR5		*P* value
	Low group, number of patients	High group, number of patients	
Age, years			
≤ 52	39	84	0.5329
> 52	45	82	
Sex			
Female	45	74	0.1787
Male	39	92	
Size (cm)			
≤ 5	51	70	0.0056
> 5	33	96	
AJCC stage			
I	27	26	0.0097
II	24	59	
III	20	63	
IV	13	18	
Histological grade			
G1	16	22	0.0013
G2	46	58	
G3	20	71	
G4	2	15	
Differentiation			
Low	25	33	0.0851
Moderate	45	88	
High	14	45	
Lung metastasis			
Yes	33	90	0.0257
No	51	76	
HBV infection			
Yes	42	93	0.3667
No	42	73	

*HBV* hepatitis B virus. Differences between groups were determined by chi-square test

### UBR5 silencing promotes apoptosis and inhibits glycolysis and AXIN1 ubiquitination in HepG2 cells

**Fig. 2:**
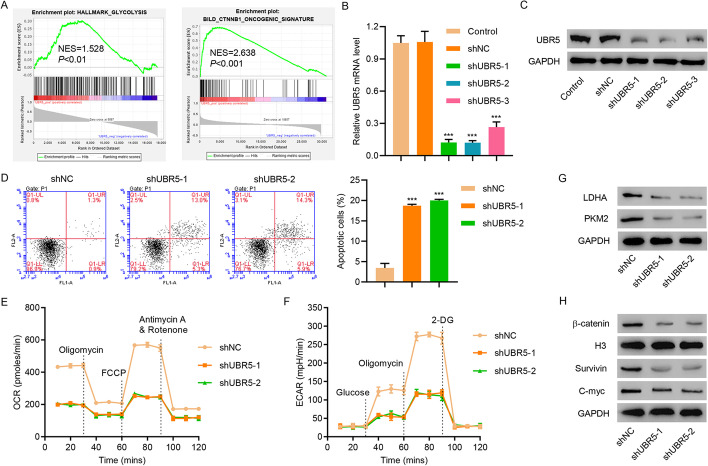
UBR5 knockdown induces cell apoptosis and reduces OCR and ECAR levels in HepG2 cells. **A** GSEA showed that UBR5 expression is associated with glycolysis and β-catenin signaling. **B**, **C** UBR5 expression, **D** cell apoptosis, **E** OCR, **F** ECAR, and **G**, **H** LDHA, PKM2, Survivin, C-myc, and β-catenin expression in HepG2 cells transduced with indicated plasmids. ***P < 0.001 compared with shNC

GSEA showed that UBR5 expression was associated with upregulation of glycolysis and the β-catenin signaling pathway (Fig. 2A). All three shRNAs specifically targeting UBR5 efficiently downregulated UBR5 expression in HepG2 cells (Fig. 2B and C), which demonstrated higher UBR5 expression compared with LO2 and Huh7 cells (Additional file 1: Fig. S1B). A decrease in UBR5 expression promoted HepG2 cell apoptosis (Fig. 2D). In addition, UBR5 knockdown decreased the OCR and ECAR (Fig. 2E and F), which are indicators of mitochondrial respiration and glycolysis, and significantly decreased LDHA, PKM2, β-catenin, C-myc, and Survivin levels in HepG2 cells (Fig. 2G and H).

AXIN1 is a rate‐limiting protein in the β‐catenin destruction complex, and its stability is controlled by the ubiquitin–proteasome system [30]. Moreover, evidence suggests that AXIN1 is also a substrate for UBR5 by UbiBrowser. To examine the interaction between AXIN1 and UBR5, co-immunoprecipitation was performed in HepG2 cells. As shown in Additional file 1: Fig. S2A, UBR5 co-immunoprecipitated with AXIN1. Reciprocal immunoprecipitation with AXIN1 antibodies also brought down UBR5, suggesting an interaction between UBR5 and AXIN1. UBR5 overexpression did not change AXIN1 mRNA levels but significantly decreased AXIN1 protein levels (Additional file 1: Fig. S2B). UBR5 silencing inhibited AXIN1 ubiquitination in HepG2 cells (Additional file 1: Fig. S2C). In addition, AXIN1 overexpression inhibited UBR5 overexpression-induced β-catenin activation in HepG2 cells (Additional file 1: Fig. S2D). These results showed that UBR5 regulated β-catenin signaling via AXIN1 ubiquitination in HCC.

### Characterizations of nanoparticles

**Fig. 3:**
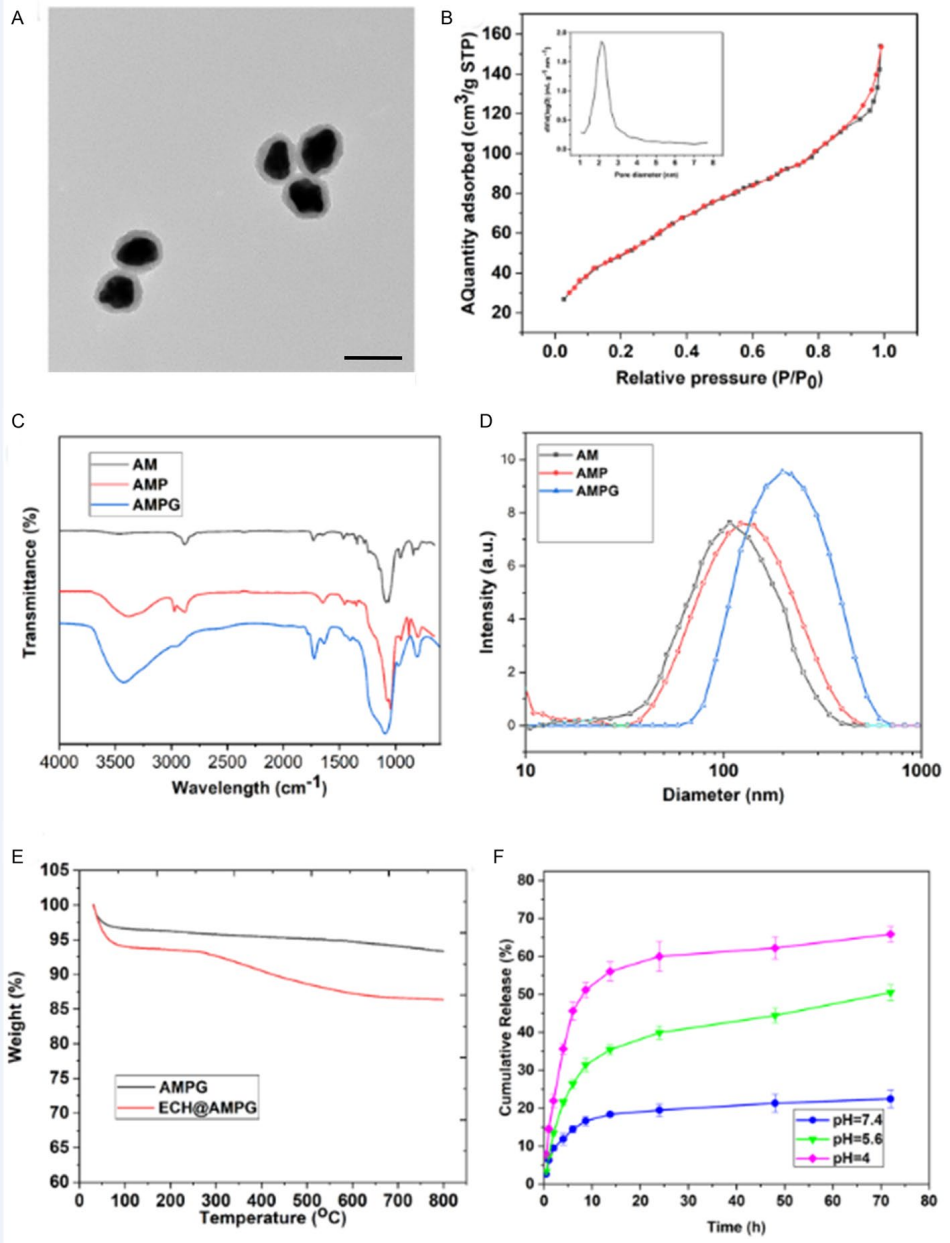
AM characterization. **A** TEM image of AM. Scale bar, 100 nm. **B** N_2_ adsorption–desorption isotherms of AM. **C** FT-IR images of AM, AMP, and AMPG. **D** DLS particle size distribution of AM, AMP, and AMPG. **E** TGA isotherms of AMPG and ECH@AMPG. **F** Release profile of ECH under different pH conditions in vitro

The AM diameter was ~ 80 nm with a mesoporous silica shell (Fig. 3A), the AM mesopore thickness was ~ 20 nm, and the AM core diameter was ~ 60 nm. A characteristic type IV curve constructed from the N_2_ adsorption –desorption isotherms of AM further confirmed their apparent mesoporous structure and narrow pore distribution (mean pore diameter 2.6 nm) (Fig. 3B). The mesoporous structure boosted the AM surface area to 486.25 m^2^/g, which could enhance the likelihood of conjugation to ECH. The characteristic C–H vibration bands at 2850–2930 cm^−1^ disappeared after the extraction of CTAB surfactants (Fig. 3C). However, AMP showed a C–H mode at 2850–2950 cm^−1^ reinforcement, which was due to the C–H bonds in the PEGDE block (Fig. 3C). After additional GAL grafting, an evident infrared peak centered at 1412 cm^−1^ appeared, which was due to the N–H vibration mode [31] (Fig. 3C), indicating that GAL was successfully introduced to the NP surface. The DLS NP size distribution also confirmed that the AM size was ~ 80 nm (Fig. 3D), which slightly improved with PEGDE grafting (AMP). In addition, a diameter leap was observed between the AM and AMPG (hydrated ionic diameter ~ 200 nm), indicating that AMPG groups were successfully grafted on the AM surface. TGA showed that NPs had a loading capacity of 8.5 wt%; thus, the proportion of conjugated ECH was ~ 85 mg/g (Fig. 3E). In addition, the release profile of ECH from ECH@AMPG was performed at pH values of 4, 5.6, and 7.4, which resembled the physiological pH conditions in cancer cell endosome, lysosome, and normal cellular environments, respectively. We observed a 70-h sustained release profile of ECH from ECH@AMPG at different pH values (Fig. 3F). At a comparatively low pH of 4, ~ 65% of the ECH was discharged from the nanovehicles in 70 h. In contrast, at a physiological pH of 7.4, only 20% of the ECH was discharged in the same duration. Strong acidity augmented the ECH discharge for the first 12 h (Fig. 3F).

### Effects of ECH@AMPG on apoptosis and glycolysis in HepG2 cells

**Fig. 4:**
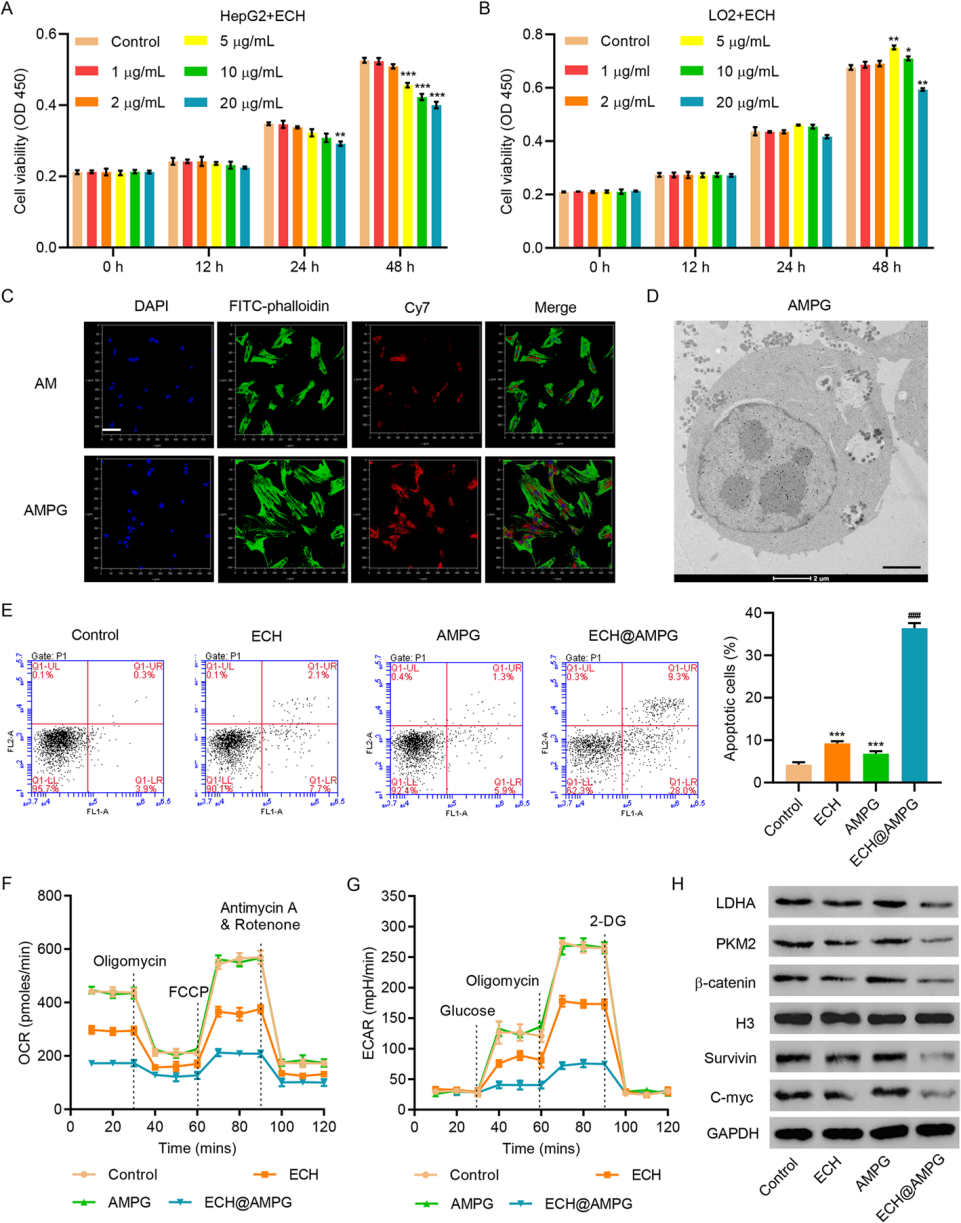
AMPG enhances the anticancer effects of ECH in HepG2 cells. Cell viability of (**A**) HepG2 and (**B**) LO2 cells incubated with different concentrations of ECH for 0, 12, 24, and 48 h. **C** CLSM images (blue: nuclei stained with DAPI; red: Cy7-labeled AM; green: cytoskeleton stained with FITC–phalloidin). Scale bars, 100 μm. **D** TEM images showing intracellular distribution of AMPG in HepG2 cells. Scale bars, 2 μm. **E** Cell apoptosis, **F** OCR, **G** ECAR, and **I** UBR5, LDHA, PKM2, Survivin, C-myc, and β-catenin expression in HepG2 cells incubated with ECH, AMPG, and ECH@AMPG. *P < 0.05, **P < 0.01, ***P < 0.001 compared with control. ^###^*P* < 0.001 compared to ECH

More than 90% of HepG2 and LO2 cells treated with AMPG nanocomposites for 24 and 48 h survived and withstood concentrations up to 5 μg/mL, indicating that AMPG had low cytotoxicity and minimal side effects on the cells (data not shown) and promising biological safety. After incubation with free ECH, HepG2 cell viability decreased steadily to 86.7% at an ECH concentration of 5 μg/mL at 48 h (Fig. 4A). However, LO2 cells incubated with free ECH at an ECH concentration of 5 μg/mL for 48 h had significantly higher cell viability (Fig. 4B). These results showed that ECH is toxic to tumor cells but not normal cells. The AMPG uptake was more obvious than the AM uptake (Fig. 4C). TEM examination of HepG2 cells incubated with AMPG for 6 h verified the integration of AMPG and HepG2 cells (Fig. 4D).

We observed no significant difference in HepG2 cell apoptosis and OCR and ECAR between blank controls and HepG2 cells incubated with AMPG (Fig. 4E–G). Parallel with blank controls and HepG2 cells incubated with AMPG, HepG2 cells treated with 5 μg/mL free ECH or ECH@AMPG had significantly increased cell apoptosis and decreased OCR and ECAR (Fig. 4E–G). These results showed that AMPG enhances the effects of ECH against HepG2 cells. ECH treatment significantly decreased UBR5 expression in HepG2 cells (Additional file 1: Fig. S3). We also observed the inhibitory effects of ECH on LDHA, PKM2, β-catenin, Survivin, and C-myc expression, especially in ECH@AMPG-treated HepG2 cells (Fig. 4H).

### Effects of ECH@AMPG on UBR5 overexpression-mediated apoptosis and glycolysis in HepG2 cells

**Fig. 5:**
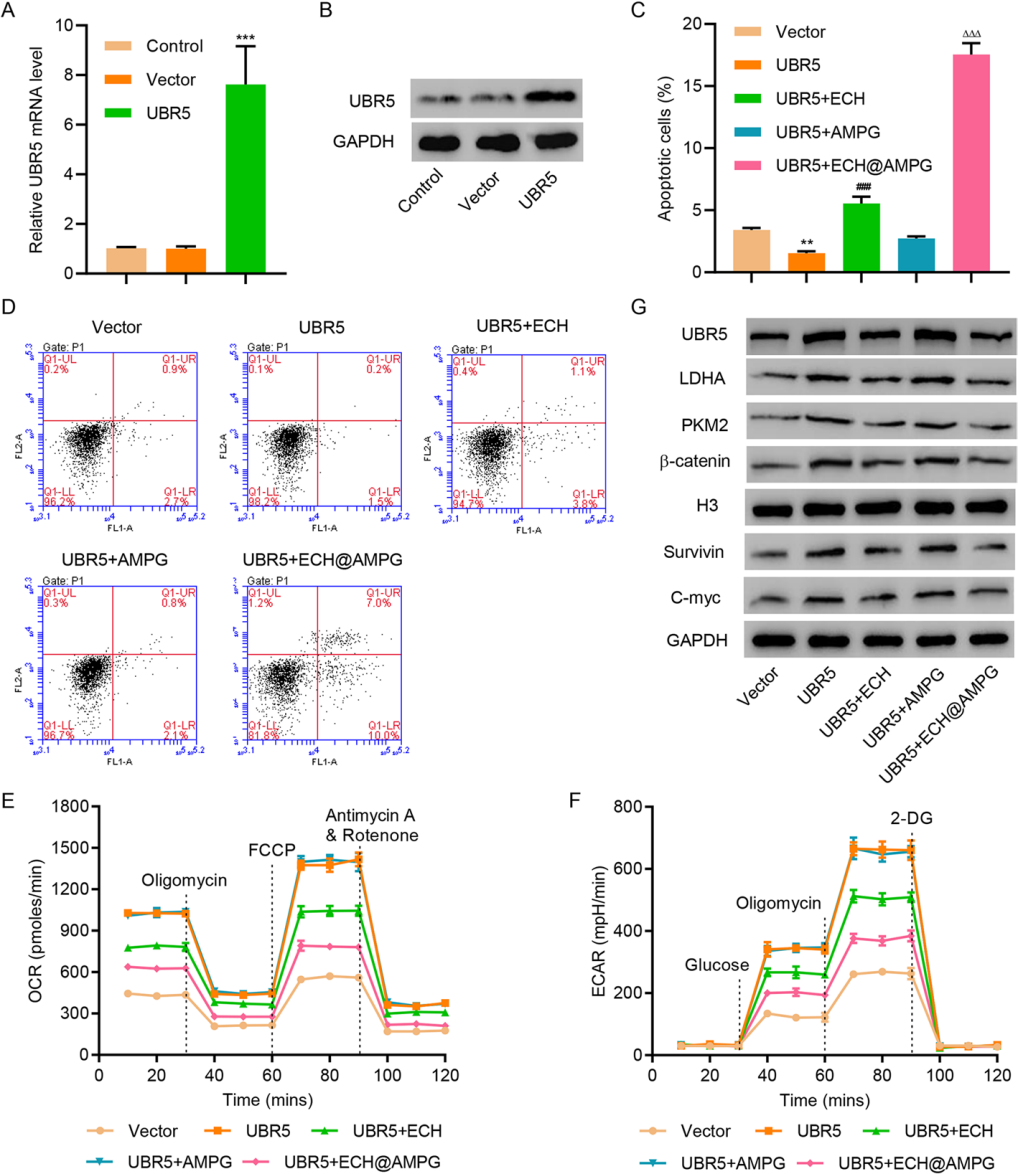
AMPG enhances the anticancer effects of ECH in HepG2 cells with UBR5 overexpression. **A**, **B** mRNA and protein expression of UBR5 in HepG2 cells transduced with indicated plasmids. **C**, **D** Cell apoptosis, **E** OCR, **F** ECAR, and **G** UBR5, LDHA, PKM2, Survivin, C-myc, and β-catenin expression in HepG2 cells transduced with indicated plasmids and incubated with ECH, AMPG, and ECH@AMPG. **P < 0.01, ***P < 0.001 compared with vector. ^###^*P* < 0.001 compared with UBR5. ^ΔΔΔ^*P* < 0.001 compared with UBR5 + ECH

UBR5 overexpression decreased apoptosis and increased the OCR and ECAR of HepG2 cells (Fig. 5A–F). In addition, UBR5 overexpression increased LDHA, PKM2, β-catenin, Survivin, and C-myc expression in HepG2 cells (Fig. 5G). However, ECH treatment significantly decreased the effects of UBR5 overexpression in HepG2 cells, especially in ECH@AMPG-treated HepG2 cells (Fig. 5C–G).

### Pharmacokinetics and the targeting effect of AMPG in vivo

**Fig. 6:**
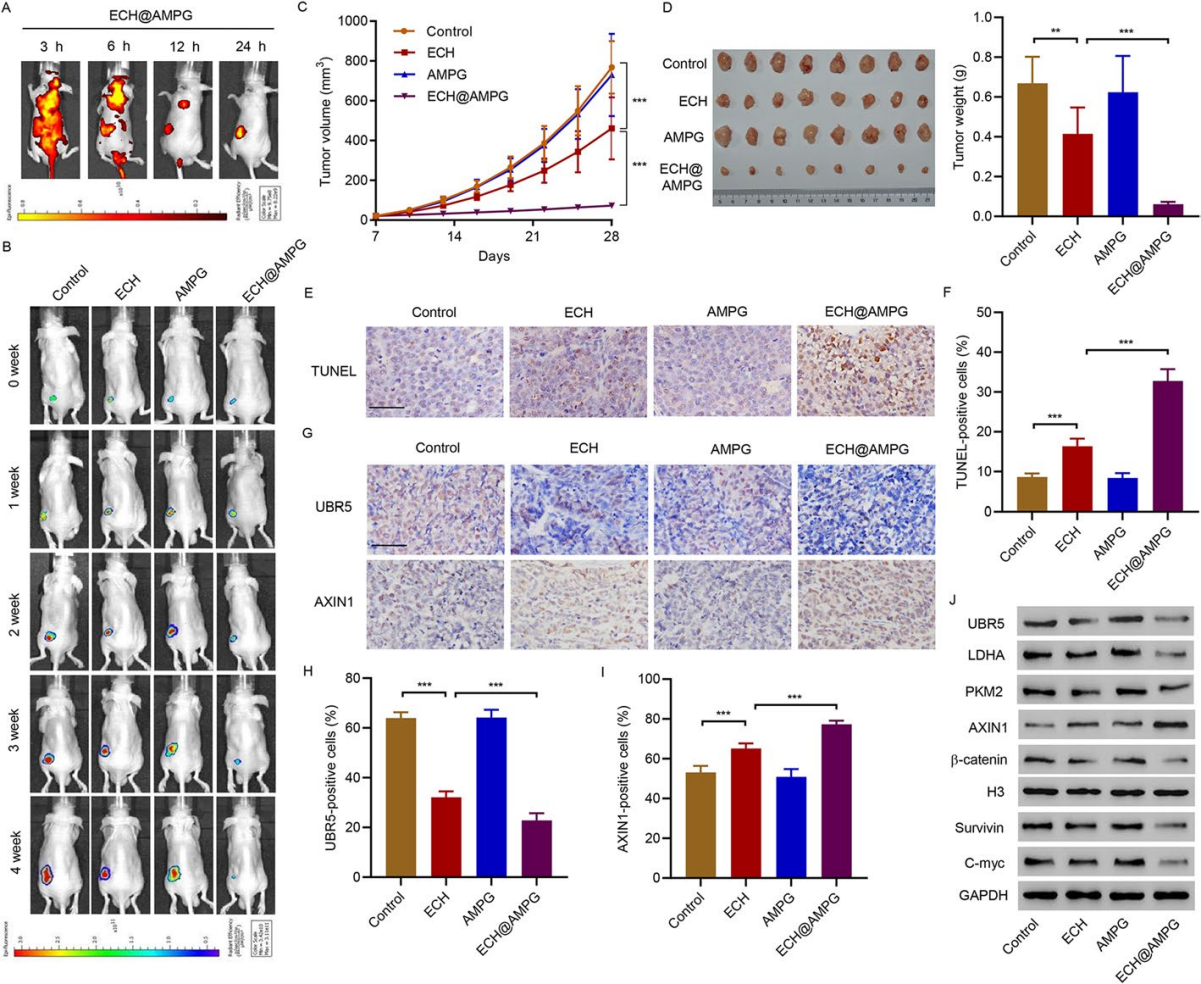
Antitumor efficacy of AMPG in HCC mouse model bearing HepG2 cells. **A** Representative fluorescence (ECH@AMPG in red) in HepG2 cell subcutaneous injection-induced xenograft tumor model at 3, 6, 12, and 24 h after intravenous injection of ECH@AMPG (5 mg/kg/day). **B**–**J** Xenograft mice 4 weeks post-injection with ECH (5 mg/kg/day), AMPG (5 mg/kg/day), or ECH@AMPG (5 mg/kg/day). **B** HepG2 cell survival. Tumor (**C**) volume and (**D**) weight. **E**, **F** TUNEL-positive cells. **G**–**I** IHC staining of UBR5 and AXIN1. **J** Xenograft mouse tumors showing UBR5, AXIN1, LDHA, PKM2, Survivin, C-myc, and β-catenin expression. Scale bars, 50 μm. **P < 0.01, ***P < 0.001

After intravenous injection of ECH@AMPG, a fluorescent (red) signal was observed for AMPG at indicated times (Fig. 6A). H&E staining showed no significant histopathological differences in the spleen, liver, heart, kidney, and lung tissue samples among all groups (Additional file 1: Fig. S4). Figure 6B shows the HepG2 survival in mice treated with ECH, AMPG, and ECH@AMPG. Compared with free ECH, ECH@AMPG administration significantly decreased tumor volume (Fig. 6C) and tumor weight (Fig. 6D). TUNEL analysis showed increased HepG2 cell apoptosis in ECH-treated mice (Fig. 6E and F). ECH inhibited UBR5, LDHA, PKM2, β-catenin, Survivin, and C-myc expression and increased AXIN1 expression in mice (Fig. 6G–J). Importantly, AMPG enhanced the inhibitory effects of ECH in HepG2 cell-bearing mice (Fig. 6B–J). Overall, consistent with our in vitro results, ECH@AMPG demonstrated efficacious therapeutic potential in vivo with minor systemic toxicity.

## Discussion

Ubiquitin protein ligase E3 has been associated with tumorigenesis via the stabilization or degradation of target proteins [5, 32], with higher levels of ubiquitin protein ligase E3 with CNVs potentially being associated with tumorigenesis; however, the molecular mechanisms underlying the role of UBR5 in HCC remain unknown. Although several types of ubiquitin protein ligase E3 have been identified, most of them have not been comprehensively studied [24]. UBR5 amplification could be a basis for UBR5 overexpression, commonly observed in HCC, and both its amplification and protein levels could be prognostic biomarkers in HCC. Glycolysis and β-catenin signaling pathway-associated genes are upregulated in HCC tissues with elevated UBR5 expression, with some of them being confirmed in HCC cells. Aerobic glycolysis is a hallmark of liver cancer and contributes to cell proliferation, angiogenesis, metastasis, invasion, immune evasion, and drug resistance in HCC [33]. During glycolysis, numerous metabolites/byproducts promote the synthesis of macromolecules stimulating cell proliferation. Furthermore, some intracellular signaling pathways of glycolysis with genetic drivers influence other characteristics of cancerous cells, including apoptosis [34]. We can therefore hypothesize that UBR5 exerts anti-apoptotic effects in HCC and may be involved in antiglycolysis effects, which is consistent with the results provided in previous studies [8, 11]. β-Catenin and its downstream target C-myc can directly or indirectly induce glycolysis-regulatory molecules LDHA and PKM2 [35, 36], indicating that a similar mechanism could be involved in this case. Moreover, UBR5 stabilized β-catenin through AXIN1 ubiquitination and degradation. Therefore, we suggested that UBR5 regulates apoptosis and glycolysis of HCC cells by β-catenin signaling. 300 ubiquitin ligase genes could be regarded as probable drug targets. However, given that UBR5 might have other client proteins, such as SOX2, p38, and Bcl-2 [8, 37], and the β-catenin signaling pathway has other negative regulators, such as APC, CKI-α, and GSK-3β [38], future studies regarding other UBR5/β-catenin-regulated signaling pathways and molecules involved in HCC tumorigenesis are warranted.

Determining the mechanisms of action of natural products derived from Chinese herbs can expedite the development of safe and effective anticancer drugs. ECH has pro-apoptotic and inactive β-catenin functions [15, 17] while also acting as an antiglycolytic agent in HCC. Therefore, ECH is a potential drug candidate for HCC therapy. ECH promotes the proliferation of human renal tubular epithelial [39], non-small cell lung cancer [40], breast cancer [17], or HCC cells [15] by blocking NF‑κB, ERK, Wnt/β-catenin, or PI3K/AKT signaling pathways, which may decrease the expression of UBR5. Nonetheless, the mechanism by which ECH lowers of UBR5 should be further considered. Considering the positive correlation between UBR5 expression and glycolysis or the β-catenin signaling pathway and the inhibition of UBR5 expression by ECH in HCC, ECH may regulate HCC glycolysis and β-catenin signaling pathway by targeting UBR5. Although ECH has multiple pharmacological activities, oral administration of ECH fails to achieve its therapeutic potential, largely because of poor absorption and low bioavailability [18, 19]. In addition, side effects also play a major role in limiting the clinical application of ECH. However, the sensitivity of HepG2 cells to ECH can be improved using ECH@AMPG drug delivery system. ECH@AMPG can be a suitable drug carrier [41] and photoacoustic imaging agent [42]. It has a characteristic mesoporous structure and narrow pore distribution (mean pore diameter, 2.6 nm). In addition, it has high solubility due to a hydrated ionic diameter of ~ 200 nm [43]. With its high drug-loading capacity, ECH@AMPG can contribute to HCC therapy. Drug discharge properties are important, especially for practical drug delivery (e.g., the liver cancer chemotherapy system) [19]. The crucial drug-releasing feature of AMPG is pH sensitivity, and its acidic pH (4 and 5.6) resembles that of cancer cell endosomes/lysosomes [44], which accelerates drug release. However, sustained release of ECH from ECH@AMPG is crucial for minimizing the leakage of the encapsulated drug and shielding ECH from degradation in the physiological environment before reaching its target cells. The amount of ECH released under normal physiological pH (7.4) is low (~ 20%), which is crucial in ensuring normal cells are exposed to less drug cytotoxicity. Therefore, the amount of ECH released from AMPG was 0.255 μg/mL (lower than the 5 μg/mL observed in the current study and the 20 μg/mL reported in our previous study [15]), which can inhibit HCC cell proliferation. These results indicate that AMPG is a strategic nanocarrier that ensures the drug is more cytotoxic to cancer cells but less cytotoxic to normal cells, which is consistent with the effect of ECH@AMPG and free ECH on HepG2 and LO2 cell viability as well as the pathological changes in major organs and tumor tissues of xenograft mice. Taken together, core–shell ECH@AMPG can be used for HCC therapy. In addition, AMPG enhances the effects of ECH against HepG2 cells, as evidenced by increased cell apoptosis and decreased glycolysis and β-catenin signaling.

## Conclusions

UBR5 CNVs are prevalent and associated with poor prognosis in patients with HCC. Moreover, UBR5 expression is correlated with decreased cell apoptosis and increased glycolysis in HepG2 cells via the β-catenin signaling pathway and is inhibited by ECH. AMPG nanocomposites show low cytotoxicity and favorable biological safety to normal cells. ECH@AMPG has the potential to effectively decrease cell glycolysis and promote cell apoptosis. ECH@AMPG also has superior inhibitory effects on HCC cells compared with free ECH. Therefore, AMPG is an attractive nanocarrier that can be used to deliver ECH to target cells and can revolutionize HCC therapy.

## Supplementary Information

**Additional file 1. FigureS1. **Analysis of UBR family gene expression of HCC in the TCGAdatabase.(A) UBR1 (*P*<0.001), UBR2 (*P*<0.001), UBR3 (*P*<0.01), UBR4 (*P*<0.001), UBR5 (*P*<0.001), UBR6 (*P*<0.001), and UBR7 (*P*<0.001) expression increased in HCC tissues compared with normaltissues. (B) UBR5 expression in LO2, HepG2 and Huh7 cells. *P<0.05, ***P<0.001 compared to LO2. **FigureS2. **UBR5 activates β-cateninsignaling by inducing ubiquitination of AXIN1.(A) Interaction between UBR5 and AXIN1 in HepG2 cells. (B) UBR5 andAXIN1 expression in HepG2 cells transduced with indicated plasmids.(C) AXIN1 ubiquitination in HepG2 cells transduced with indicatedplasmids. (D) AXIN1 and β-cateninexpression in HepG2 cells transduced with indicated plasmids.***P<0.001 compared to shNC+vector. **Figure S3. **ECHinhibits UBR5 expression in HCC cells.UBR5expression in HepG2 cells incubated with different concentrations ofECH. ***P<0.001 compared to control. **FigureS4. **Imagesof H&E-stained sections obtained from key organs (liver, heart,spleen, kidneys, and lungs) from xenograft mice 4 weekspost-injection with ECH (5 mg/kg/d), AMPG (5 mg/kg/d), or ECH@AMPG (5mg/kg/d).Scalebars: 100 μm.

## Data Availability

All data presented in this study are included within the paper and its additional files.

## Abbreviations

HCC: Hepatocellular carcinoma
UBR5: Ubiquitin protein ligase E3 component N-recognin 5
ECH: Echinacoside
MSN: Mesoporous silica nanoparticle
GAL: Galactose
PEGDE: Poly(ethylene glycol) diglycidyl ether
CNV: Copy-number variation
NPs: Nanoparticles
CTAB: Cetyltrimethyl ammonium bromide
NaBH_4_: Sodium borohydride
TEOS: Tetraethyl orthosilicate
DMSO: Dimethyl sulfoxide
HAuCl_4_·3H_2_O: Tetrachloroauric acid
TCGA: The Cancer Genome Atlas
GSEA: Gene set enrichment analysis
IHC: Immunohistochemistry
TEM: Transmission electron microscopy
FT-IR: Fourier transform infrared
TGA: Thermogravimetric analysis
DLS: Dynamic light scattering
PBS: Phosphate-buffered saline
CCK-8: Cell Counting Kit 8
CLSM: Confocal laser scanning microscopy
FITC: Fluorescein isothiocyanate
DAPI: 4′,6-Diamidino-2-phenylindole
OCR: Oxygen consumption rate
ECAR: Extracellular acidification rate
qRT-PCR: Quantitative reverse-transcription polymerase chain reaction
LDHA: Lactate dehydrogenase A
PKM2: Pyruvate kinase isozymes M2
H&E: Hematoxylin and eosin
TUNEL: Terminal deoxynucleoitidyl transferase dUTP nick end labeling
ANOVA: One-way analysis of variance
AJCC: American Joint Committee on Cancer
HBV: Hepatitis B virus
