# Supplementary material for: Targeting UBR5 in hepatocellular carcinoma cells and precise treatment via echinacoside nanodelivery
Source: Cell Mol Biol Lett. 2022 Oct 12;27:92. doi: 10.1186/s11658-022-00394-w (PMC9558419; doi:10.1186/s11658-022-00394-w)
Supplement: Supplementary file 1 — Additional file 1. FigureS1. Analysis of UBR family gene expression of HCC in the TCGAdatabase.(A) UBR1 (P<0.001), UBR2 (P<0.001), UBR3 (P<0.01), UBR4 (P<0.001), UBR5 (P<0.001), UBR6 (P<0.001), and UBR7 (P<0.001) expression increased in HCC tissues compared with normaltissues. (B) UBR5 expression in LO2, HepG2 and Huh7 cells. *P<0.05, ***P<0.001 compared to LO2. FigureS2. UBR5 activates β-cateninsignaling by inducing ubiquitination of AXIN1.(A) Interaction between UBR5 and AXIN1 in HepG2 cells. (B) UBR5 andAXIN1 expression in HepG2 cells transduced with indicated plasmids.(C) AXIN1 ubiquitination in HepG2 cells transduced with indicatedplasmids. (D) AXIN1 and β-cateninexpression in HepG2 cells transduced with indicated plasmids.***P<0.001 compared to shNC+vector. Figure S3. ECHinhibits UBR5 expression in HCC cells.UBR5expression in HepG2 cells incubated with different concentrations ofECH. ***P<0.001 compared to control. FigureS4. Imagesof H&E-stained sections obtained from key organs (liver, heart,spleen, kidneys, and lungs) from xenograft mice 4 weekspost-injection with ECH (5 mg/kg/d), AMPG (5 mg/kg/d), or ECH@AMPG (5mg/kg/d).Scalebars: 100 μm. [file 11658_2022_394_MOESM1_ESM.docx]

**
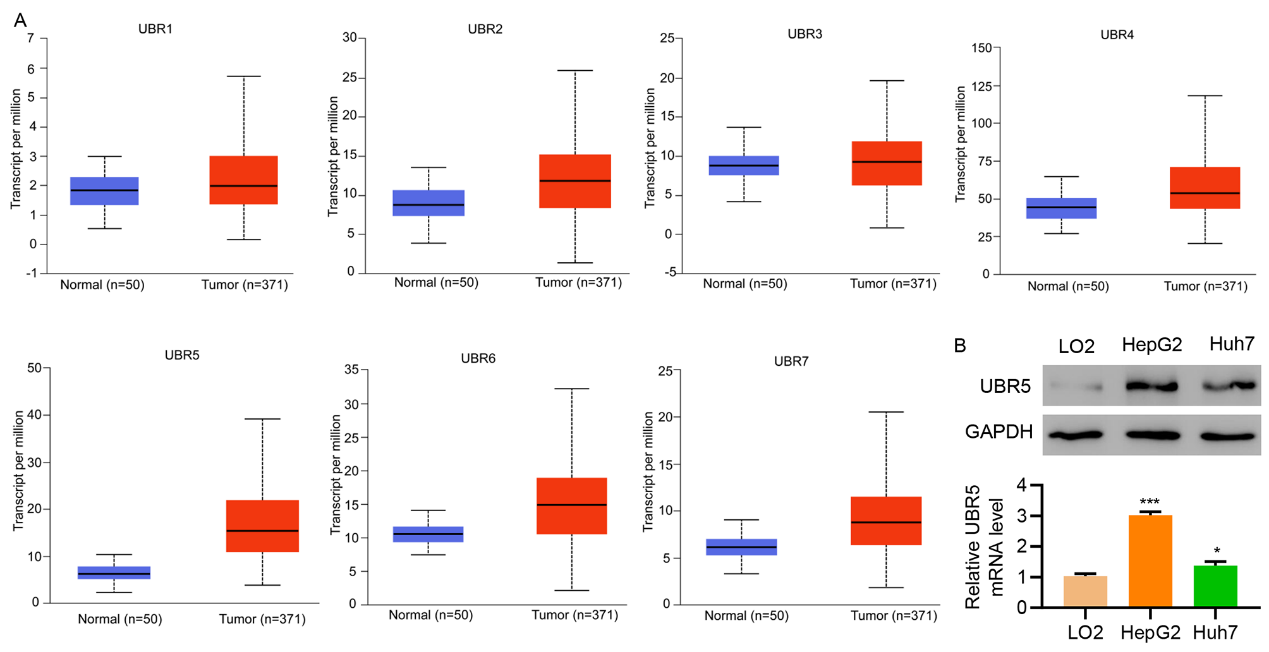
**

**Figure S1. Analysis of UBR family gene expression of HCC in the TCGA database**. (A) UBR1 (*P* < 0.001), UBR2 (*P* < 0.001), UBR3 (*P* < 0.01), UBR4 (*P* < 0.001), UBR5 (*P* < 0.001), UBR6 (*P* < 0.001), and UBR7 (*P* < 0.001) expression increased in HCC tissues compared with normal tissues. (B) UBR5 expression in LO2, HepG2 and Huh7 cells. **P* < 0.05, ****P* < 0.001 compared to LO2.


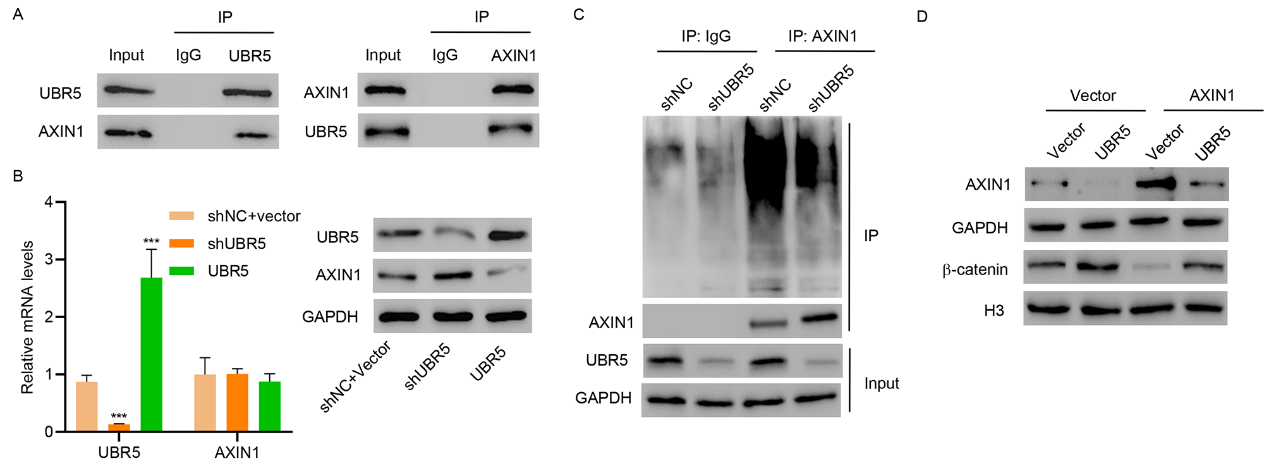


**Figure S2. UBR5 activates β-catenin signaling by inducing ubiquitination of AXIN1.** (A) Interaction between UBR5 and AXIN1 in HepG2 cells. (B) UBR5 and AXIN1 expression in HepG2 cells transduced with indicated plasmids. (C) AXIN1 ubiquitination in HepG2 cells transduced with indicated plasmids. (D) AXIN1 and β-catenin expression in HepG2 cells transduced with indicated plasmids. ****P* < 0.001 compared to shNC+vector.


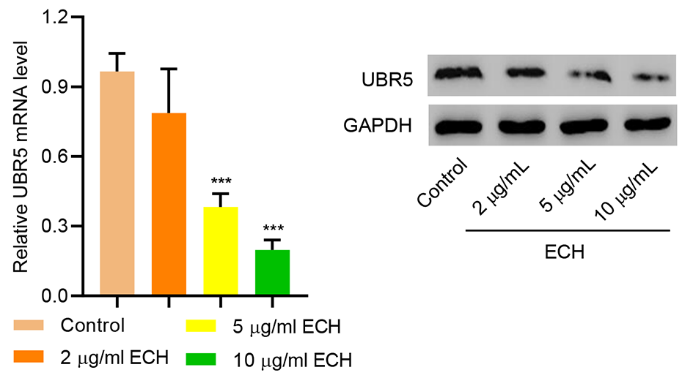


**Figure S3. ECH inhibits UBR5 expression in HCC cells.** UBR5 expression in HepG2 cells incubated with different concentrations of ECH. ****P* < 0.001 compared to control.


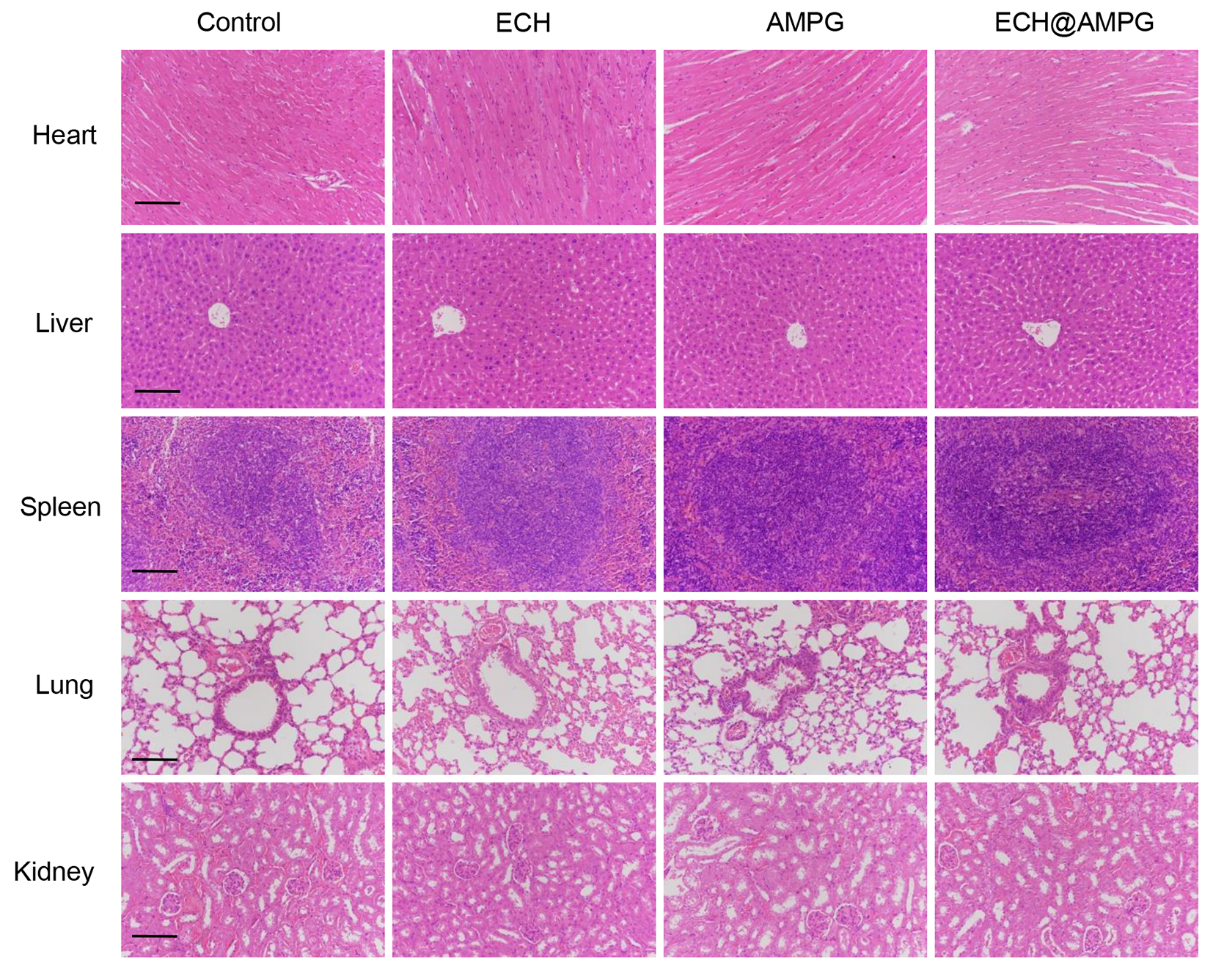


**Figure S4.** Images of H&E-stained sections obtained from key organs (liver, heart, spleen, kidneys, and lungs) from xenograft mice 4 weeks post-injection with ECH (5 mg/kg/d), AMPG (5 mg/kg/d), or ECH@AMPG (5 mg/kg/d). Scale bars: 100 μm.
